# Comparison of Symptoms Associated With SARS-CoV-2 Variants Among Children in Canada

**DOI:** 10.1001/jamanetworkopen.2023.2328

**Published:** 2023-03-09

**Authors:** Madeleine W. Sumner, Jianling Xie, Roger Zemek, Kathleen Winston, Gabrielle Freire, Brett Burstein, April Kam, Jason Emsley, Jocelyn Gravel, Robert Porter, Vikram Sabhaney, Ahmed Mater, Marina I. Salvadori, Simon Berthelot, Darcy Beer, Naveen Poonai, Anne Moffatt, Bruce Wright, Stephen B. Freedman

**Affiliations:** 1 Schulich School of Medicine and Dentistry, Western University, London, Ontario, Canada; 2 Section of Pediatric Emergency Medicine, Department of Pediatrics, Cumming School of Medicine, University of Calgary, Calgary, Alberta, Canada; 3 Department of Pediatrics, University of Ottawa, Children’s Hospital of Eastern Ontario, Ottawa, Ontario, Canada; 4 Department of Emergency Medicine, University of Ottawa, Children’s Hospital of Eastern Ontario, Ottawa, Ontario, Canada; 5 Department of Pediatrics, Cumming School of Medicine, University of Calgary, Calgary, Alberta, Canada; 6 Division of Emergency Medicine, Department of Paediatrics, Hospital for Sick Children, Faculty of Medicine, University of Toronto, Toronto, Ontario, Canada; 7 Division of Pediatric Emergency Medicine, Department of Pediatrics, Montreal Children’s Hospital, McGill University Health Centre, Montreal, Quebec, Canada; 8 Department of Epidemiology, Biostatistics, and Occupational Health, McGill University, Montreal, Quebec, Canada; 9 Division of Emergency Medicine, Department of Pediatrics, McMaster Children’s Hospital, Hamilton, Ontario, Canada; 10 Department of Emergency Medicine, IWK Children’s Health Centre and Queen Elizabeth II Health Sciences Centre, Dalhousie University, Halifax, Nova Scotia, Canada; 11 Department of Pediatric Emergency Medicine, Centre Hospitalier Universitaire (CHU) Sainte-Justine, Université de Montréal, Montreal, Quebec, Canada; 12 Janeway Children’s Health and Rehabilitation Centre, Eastern Health, St John’s, Newfoundland and Labrador, Canada; 13 Department of Paediatrics, BC Children’s Hospital, University of British Columbia, Vancouver, British Columbia, Canada; 14 Section of Pediatric Emergency, Department of Pediatrics, Jim Pattison Children’s Hospital, University of Saskatchewan, Saskatoon, Saskatchewan, Canada; 15 Public Health Agency of Canada, Ottawa, Ontario, Canada; 16 Department of Pediatrics, McGill University, Montreal, Quebec, Canada; 17 Département de Médecine Familiale et de Médecine d’Urgence, CHU de Québec-Université, Québec City, Quebec, Canada; 18 Department of Pediatrics and Child Health, The Children’s Hospital of Winnipeg, Children’s Hospital Research Institute of Manitoba, University of Manitoba, Winnipeg, Canada; 19 Department of Paediatrics, Children’s Hospital London Health Sciences Centre, Schulich School of Medicine & Dentistry, London, Ontario, Canada; 20 Department of Internal Medicine, Children’s Hospital London Health Sciences Centre, Schulich School of Medicine & Dentistry, London, Ontario, Canada; 21 Department of Epidemiology & Biostatistics, Children’s Hospital London Health Sciences Centre, Schulich School of Medicine & Dentistry, London, Ontario, Canada; 22 Department of Paediatrics, Kingston Health Sciences Centre, Queen’s University, Kingston, Ontario, Canada; 23 University of Alberta, Department of Pediatrics, Stollery Children’s Hospital, Edmonton, Alberta, Canada; 24 Sections of Pediatric Emergency Medicine and Gastroenterology, Departments of Pediatrics and Emergency Medicine, Cumming School of Medicine, University of Calgary, Calgary, Alberta, Canada

## Abstract

**Importance:**

Clinical manifestations of SARS-CoV-2 variants have not been systematically compared in children.

**Objective:**

To compare symptoms, emergency department (ED) chest radiography, treatments, and outcomes among children with different SARS-CoV-2 variants.

**Design, Setting, and Participants:**

This multicenter cohort study was performed at 14 Canadian pediatric EDs. Participants included children and adolescents younger than 18 years (hereinafter referred to as children) tested for SARS-CoV-2 infection in an ED between August 4, 2020, and February 22, 2022, with 14 days of follow-up.

**Exposure(s):**

SARS-CoV-2 variants detected on a specimen collected from the nasopharynx, nares, or throat.

**Main Outcomes and Measures:**

The primary outcome was presence and number of presenting symptoms. The secondary outcomes were presence of core COVID-19 symptoms, chest radiography findings, treatments, and 14-day outcomes.

**Results:**

Among 7272 participants presenting to an ED, 1440 (19.8%) had test results positive for SARS-CoV-2 infection. Of these, 801 (55.6%) were boys, with a median age of 2.0 (IQR, 0.6-7.0) years. Children with the Alpha variant reported the fewest core COVID-19 symptoms (195 of 237 [82.3%]), which were most often reported by participants with Omicron variant infection (434 of 468 [92.7%]; difference, 10.5% [95% CI, 5.1%-15.9%]). In a multivariable model with the original type as the referent, the Omicron and Delta variants were associated with fever (odds ratios [ORs], 2.00 [95% CI, 1.43-2.80] and 1.93 [95% CI, 1.33-2.78], respectively) and cough (ORs, 1.42 [95% CI, 1.06-1.91] and 1.57 [95% CI, 1.13-2.17], respectively). Upper respiratory tract symptoms were associated with Delta infection (OR, 1.96 [95% CI, 1.38-2.79]); lower respiratory tract and systemic symptoms were associated with Omicron variant infection (ORs, 1.42 [95% CI, 1.04-1.92] and 1.77 [95% CI, 1.24-2.52], respectively). Children with Omicron infection most often had chest radiography performed and received treatments; compared with those who had Delta infection, they were more likely to have chest radiography performed (difference, 9.7% [95% CI, 4.7%-14.8%]), to receive intravenous fluids (difference, 5.6% [95% CI, 1.0%-10.2%]) and corticosteroids (difference, 7.9% [95% CI, 3.2%-12.7%]), and to have an ED revisit (difference, 8.8% [95% CI, 3.5%-14.1%]). The proportions of children admitted to the hospital and intensive care unit did not differ between variants.

**Conclusions and Relevance:**

The findings of this cohort study of SARS-CoV-2 variants suggest that the Omicron and Delta variants were more strongly associated with fever and cough than the original-type virus and the Alpha variant. Children with Omicron variant infection were more likely to report lower respiratory tract symptoms and systemic manifestations, undergo chest radiography, and receive interventions. No differences were found in undesirable outcomes (ie, hospitalization, intensive care unit admission) across variants.

## Key Points

**Question:**

Do the symptom profiles among children with SARS-CoV-2 infection evaluated in tertiary care emergency departments differ by variant type?

**Findings:**

In this multicenter Canadian cohort study of 1440 children with SARS-CoV-2 infection, those with Alpha variant infection reported the fewest core COVID-19 symptoms, while those with Omicron variant infection reported more fever, lower respiratory tract, and systemic symptoms than those infected by other variants. Hospitalization and intensive care admission rates were comparable across variants.

**Meaning:**

Although the characteristics of presenting symptoms have changed as the SARS-CoV-2 virus has evolved, the proportions of infected children experiencing undesirable outcomes has remained stable.

## Teaser

This cohort study quantifies and compares symptoms, emergency department chest radiography, treatments, and disposition across dominant SARS-CoV-2 variants among children presenting to emergency departments in Canada.

## Introduction

The emergence of variants of concern (VoC) with varying transmissibility patterns has influenced the course of the COVID-19 pandemic.^1,2,3^ The World Health Organization has identified 5 VoC,^4^ beginning with the Alpha variant (B.1.1.7), which was detected in the United Kingdom in the fall of 2020.^5^ Each VoC has affected the pandemic uniquely, reflecting different transmissibility and clinical characteristics. The Delta variant (B.1.617.2), which was designated a VoC in May 2021,^6^ became the dominant strain worldwide in the summer and fall of that year and has been reported as more likely to lead to severe outcomes.^7^ The Omicron variant (B.1.1.529), which was identified as a VoC in November 2021,^8^ led to the largest wave of the pandemic in many countries at the end of 2021^9^ and has been notable for its high transmissibility.^10^

As the SARS-CoV-2 virus has evolved, so have the symptoms and severity of disease.^11,12^ The most recent VoC, Omicron, has a propensity to infect the upper airways,^13^ replicating faster in the bronchus than any other variants but less so in the lung parenchyma.^14^ Among adults, Omicron infection symptoms have differed from those associated with the Delta variant,^11^ and mortality is lower.^12^ Although Omicron infection in children has been associated with croup and upper airway disease,^15,16^ no reports have compared symptom prevalence starting with the original-type strain to the latest VoC, and little is known about disease severity. Thus, we sought to quantify and compare symptoms, emergency department (ED) chest radiography, treatments, and disposition across dominant SARS-CoV-2 variants in a prospective ED-based cohort study in Canada.

## Methods

### Study Design and Setting

This prospective observational cohort study recruited children and adolescents (hereinafter referred to as children) tested for acute SARS-CoV-2 infection who presented for care at 1 of 14 Canadian urban pediatric EDs that are members of the Pediatric Emergency Research Canada network^17^ between August 4, 2020, and February 22, 2022. We report data (database exported January 4, 2023) in accordance with the Strengthening the Reporting of Observational Studies in Epidemiology (STROBE) reporting guideline in relation to the index ED visit and 14-day follow-up. Research ethics board approval was obtained at all participating institutions, and the caregivers and/or guardians of all participants provided informed consent along with participant assent as required by institutional policy.

### Participants and Recruitment

Eligible participants were younger than 18 years and had a positive SARS-CoV-2 nucleic acid test result on a specimen collected from the nasopharynx, nares, or throat. As the study relied on clinical specimens, the protocol did not specify SARS-CoV-2 testing criteria or laboratory methodologies, both of which were modified by local policies as the pandemic evolved. Children were excluded if they were diagnosed as having Kawasaki disease or the multisystem inflammatory syndrome of children and if they only underwent antibody testing given the low sensitivity of these tests during acute illness^18^ and the inability to specify variants.

Each day, designated research team members received a list of all children who underwent SARS-CoV-2 nucleic acid testing in the ED. Research assistants contacted all potentially eligible children by telephone, starting with the first child tested each day, to minimize the potential for selection bias and to standardize the approach to participant recruitment.

### Outcomes

Our primary outcome was the presence and number of presenting symptoms (Table 1) from illness onset until study enrollment. Symptom groups included gastrointestinal, hydration, lower respiratory, musculoskeletal, neurological, rash or oral changes, systemic, and upper respiratory. Anosmia or ageusia, cough, conjunctivitis, and fever were analyzed independently without being grouped with other symptoms. Secondary outcomes included (1) presence of core COVID-19 symptoms (≥1 of ageusia, anosmia, cough, or fever)^19^; (2) chest radiography performance and treatment provided; and (3) hospitalization, intensive care unit (ICU) admission, and revisits to any ED or to any health care provider (including the ED) within 14 days of the index ED visit.

**Table 1.** Symptom Profile of SARS-CoV-2–Infected Children Reported by SARS-CoV-2 Variant Identified

Characteristic	All patients (N = 1440)	SARS-CoV-2 variant^a^				*P* value
		Original type (n = 409)	Alpha (n = 242)	Delta (n = 321)	Omicron (n = 468)	
Age, median (IQR), y	2.0 (0.6-7.0)	1.3 (0.6-6.0)	2.0 (0.9-7.0)	3.0 (0.9-8.0)	1.0 (0.3-6.0)	<.001
Sex, No. (%)						
Boys	801 (55.6)	227 (55.5)	148 (61.2)	176 (54.8)	250 (53.4)	<.001
Girls	639 (44.4)	182 (44.5)	94 (38.8)	145 (45.2)	218 (46.6)	
Duration of illness, median (IQR), d	3.0 (1.0-4.0)	2.0 (1.0-4.0)	2.0 (0.8-4.0)	3.0 (2.0-5.0)	3.0 (1.0-4.0)	<.001
Symptom, No. (%)						
Fever	1077 (75.1)	290 (70.9)	156 (65.8)	252 (78.5)	379 (81.0)	<.001
Cough	811 (56.6)	211 (51.6)	129 (54.4)	197 (61.4)	274 (58.8)	.07
Conjunctivitis	88 (6.1)	31 (7.6)	7 (3.0)	32 (10.0)	18 (3.9)	.003
Any upper respiratory tract	876 (61.1)	241 (58.9)	119 (50.2)	228 (71.0)	288 (61.8)	<.001
Rhinorrhea	770 (53.8)	215 (52.6)	101 (42.6)	198 (61.7)	256 (54.9)	<.001
Sore throat	315 (22.0)	76 (18.6)	38 (16.0)	91 (28.3)	110 (23.6)	.004
Any lower respiratory tract	545 (38.0)	145 (35.5)	85 (35.9)	112 (34.9)	203 (43.6)	.07
Chest pain	104 (7.3)	38 (9.3)	19 (8.0)	23 (7.2)	24 (5.2)	.19
Chest pain (pleuritic)	40 (2.8)	14 (3.4)	6 (2.5)	6 (1.9)	14 (3.0)	.75
Shortness of breath	363 (25.3)	88 (21.5)	53 (22.4)	68 (21.2)	154 (33.0)	<.001
Sputum production	161 (11.2)	38 (9.3)	22 (9.3)	40 (12.5)	61 (13.1)	.28
Wheezing	207 (14.4)	55 (13.4)	29 (12.2)	44 (13.7)	79 (17.0)	.37
Any gastrointestinal tract	906 (63.2)	272 (66.5)	146 (61.6)	201 (62.6)	287 (61.6)	.54
Abdominal pain	313 (21.8)	109 (26.7)	50 (21.1)	62 (19.3)	92 (19.7)	.09
Anorexia^b^	550 (38.4)	155 (37.9)	95 (40.1)	130 (40.5)	170 (36.5)	.75
Diarrhea	310 (21.6)	107 (26.2)	48 (20.3)	63 (19.6)	92 (19.7)	.12
Vomiting	377 (26.3)	114 (27.9)	62 (26.2)	80 (24.9)	121 (26.0)	.85
Any neurological	363 (25.3)	100 (24.4)	58 (24.5)	99 (30.8)	106 (22.7)	.12
Headache	333 (23.2)	93 (22.7)	53 (22.4)	89 (27.7)	98 (21.0)	.24
Seizures	41 (2.9)	10 (2.4)	6 (2.5)	12 (3.7)	13 (2.8)	.79
Any musculoskeletal	226 (15.8)	83 (20.3)	33 (13.9)	55 (17.1)	55 (11.8)	.01
Joint pain	149 (10.4)	50 (12.2)	22 (9.3)	42 (13.1)	35 (7.5)	.07
Myalgia	211 (14.7)	80 (19.6)	33 (13.9)	49 (15.3)	49 (10.5)	.01
Any dehydration	452 (31.5)	126 (30.8)	76 (32.1)	95 (29.6)	155 (33.3)	.79
Decreased urine output	237 (16.5)	69 (16.9)	38 (16.0)	61 (19.0)	69 (14.8)	.58
Refusal to drink^c^	379 (26.4)	102 (24.9)	64 (27.0)	74 (23.1)	139 (29.8)	.23
Any systemic	937 (65.4)	273 (66.7)	136 (57.4)	199 (62.0)	329 (70.6)	.01
Apnea^d^	16 (1.1)	4 (1.0)	4 (1.7)	4 (1.2)	4 (0.9)	.79
Drowsiness	555 (38.8)	147 (35.9)	77 (32.5)	119 (37.2)	212 (45.5)	.01
Irritable	630 (44.0)	194 (47.4)	97 (40.9)	126 (39.4)	213 (45.7)	.16
Lethargic	593 (41.4)	174 (42.5)	86 (36.3)	120 (37.4)	213 (45.7)	.07
Any rash or oral changes	290 (20.2)	105 (25.7)	43 (18.1)	58 (18.1)	84 (18.0)	.04
Extremity changes	26 (1.8)	8 (2.0)	4 (1.7)	5 (1.6)	9 (1.9)	.99
Oral changes	144 (10.0)	56 (13.7)	12 (5.1)	29 (9.0)	47 (10.1)	.01
Rash to hands	27 (1.9)	10 (2.4)	4 (1.7)	4 (1.2)	9 (1.9)	.79
Rash to feet	34 (2.4)	14 (3.4)	9 (3.8)	4 (1.3)	7 (1.5)	.12
Skin rash	144 (10.0)	52 (12.7)	25 (10.5)	28 (8.7)	39 (8.4)	.22
Anosmia	45 (3.1)	21 (5.1)	7 (3.0)	14 (4.4)	3 (0.6)	<.001
Ageusia	69 (4.8)	30 (7.3)	11 (4.6)	22 (6.9)	6 (1.3)	<.001
No. of symptoms at index visit, median (IQR)	6.0 (3.0-9.0)	6.0 (4.0-9.0)	5.0 (3.0-8.0)	6.0 (3.0-9.0)	6.0 (3.0-9.0)	.06
Core COVID-19 symptoms, No. (%)^e^	1277 (89.0)	354 (86.6)	195 (82.3)	294 (91.6)	434 (92.7)	<.001
Codetection of a respiratory virus, No./total No. (%)	35/232 (15.1)	2/63 (3.2)	3/30 (10.0)	14/35 (40.0)	16/104 (15.4)	<.001

^a^ Five patients in the Alpha group, 2 patients in the Omicron group, and 1 patient in the Delta group had missing data in at least 1 of the clinical symptoms.

^b^ Defined as less than 50% of usual food intake in the past 12 hours.

^c^ Defined as less than 50% of usual fluid intake in the past 12 hours.

^d^ Defined as no respiration over a period of 15 or more seconds.

^e^ Indicates 1 or more of fever, cough, and/or loss of smell or taste.

### Data Collection

Demographic, epidemiological, chronic conditions and comorbidities, and clinical data were collected by telephone survey from caregivers a median of 2 (IQR, 1-3) days following the ED visit. The presence of each symptom included in our study was asked about specifically as a dichotomous variable. Data were extracted from the medical record by trained study personnel regarding chest radiography and treatments. At 14-day follow-up, we collected information regarding subsequent health care visits. Collection of VoC test results was implemented on March 25, 2021, with sites retrospectively reporting available VoC results for participants enrolled prior to that date. Study procedures were standardized using a manual of operations. Data quality rules were programmed within REDCap,^20,21^ with data quality checks performed by the study statisticians (including J.X.). Queries were issued for key variables that were missing, out of range, or discrepant.

### VoC Classification

Testing and reporting of VoC varied by institution and over time. When a VoC or a mutation linked to a VoC was identified, that report was used for classification purposes. When VoC testing was not performed or results were inconclusive, the SARS-CoV-2 virus detected was classified based on the dominant strain on the test date. SARS-CoV-2 national epidemiological data were used to identify the dominant strain,^12,22^ which was defined by the variant comprising at least 50% of reported cases in Canada on a given date. This led to the following date ranges for VoC dominance periods: original strain, prior to April 18, 2021; Alpha, April 18 to June 26, 2021; Delta, June 27 to December 11, 2021; and Omicron, December 12, 2021, to February 22, 2022. Between June 13 and 26, 2021, no variant exceeded 50% of cases; as the Alpha variant was most prevalent during this period (42.8% of cases), missing variant data during that interval were classified as Alpha. The Beta, Eta, and Gamma variants never assumed dominance (ie, >50% of cases), and their peak proportion of cases were 2.6% for Beta (week of April 4, 2021); 4.8% for Eta (week of March 28, 2021); and 26.3% for Gamma (week of April 4, 2021).^22^ Given the small number of participants with these variants in our cohort (1 with Beta, 0 with Eta, and 6 with Gamma), these VoC were excluded from our analysis.

### Definitions

The original-type SARS-CoV-2 virus refers to the genomic sequence of SARS-CoV-2 identified from the first cases before the emergence of VoC. Symptoms reported by caregivers were those that were new and/or associated with the current illness. Core COVID-19 symptoms included ageusia, anosmia, cough, or fever,^19^ defined as a documented temperature of at least 38.0°C in the ED, in the referring clinic or hospital, at home, or a tactile fever.^23^

### Sample Size

Data were shared in real-time with the Public Health Agency of Canada for pandemic surveillance. Therefore, no formal sample size calculations were performed in relation to this analysis.

### Statistical Analysis

Data were summarized using counts and percentages for categorical variables and median and IQR for continuous variables. For the primary outcomes, we used mixed-effect binary logistic regression models to assess the adjusted odds ratio (OR) of experiencing a given symptom for each VoC, with the original type serving as the reference group. Fixed effects included VoC, sex, age, number of days of illness, and hospitalization at the index ED visit or within 14 days. We specified intercept-only random effects to account for correlation between individuals within the same study site. For the secondary outcomes among VoC group comparisons, we used the Kruskal-Wallis test for continuous variables and χ^2^ and Fisher exact tests for categorical variables as appropriate. Differences between proportions were estimated using a 2-sample proportion test. Given the high number of participants who had their VoC inferred, we compared the tested and inferred groups based on the method of variant assignment. All *P* values were adjusted for multiple comparisons via the Benjamini-Hochberg method,^24^ and 2-sided *P* < .05 was considered statistically significant. Analyses were performed with SPSS Statistics for Windows, version 26.0 (IBM Corporation).

## Results

### Participant Characteristics

**Figure 1.**
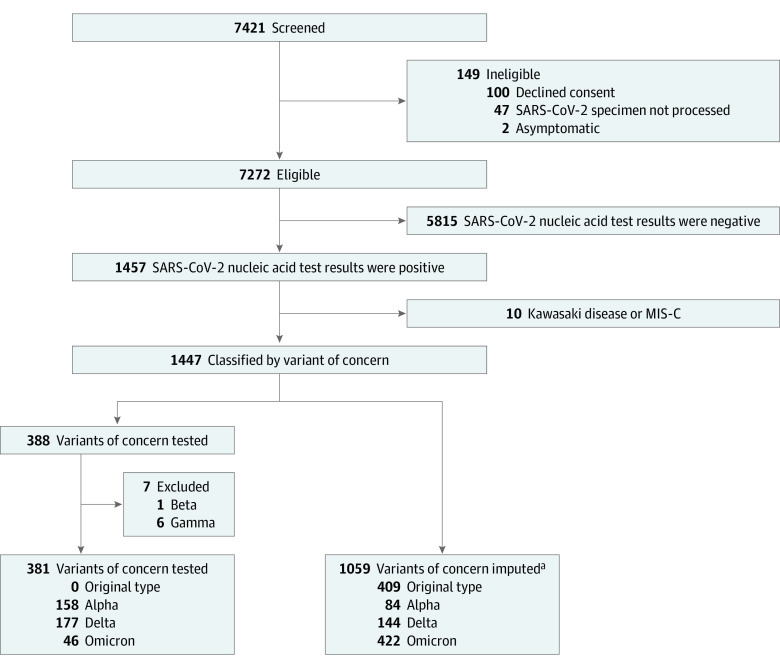
Flow Diagram of Enrolled Participants MIS-C indicates multisystem inflammatory syndrome of children. ^a^Includes 116 specimens tested for a variant of concern for which the report did not specify the lineage or the result was considered inconclusive. The imputation approach was based on the dominant variant in Canada (50% of reported cases) at the time of SARS-CoV-2 testing. Original type: before April 18, 2021; Alpha: April 18, 2021, to June 26, 2021; Delta: June 27, 2021, to December 11, 2021; Omicron: December 12, 2021, to February 22, 2022.

Of 7272 eligible patients, 1440 (19.8%) had positive test results for SARS-CoV-2 infection (Figure 1). Among the 1440 study participants, 801 (55.6%) were boys and 639 (44.4%) were girls; the median participant age was 2.0 (IQR, 0.6-7.0) years (Table 1). Owing to hundreds of possible combinations, race and ethnicity data were collected but not reported. Testing for VoC was performed in 388 participants (26.9%), which identified 158 (40.7%) Alpha, 177 (45.6%) Delta, and 46 (11.9%) Omicron variants (in addition to 1 Beta and 6 Gamma variants); the results from an additional 116 participants were inconclusive or did not specify the lineage, and their etiologic strain required imputation (eFigure 1 in Supplement 1). Concomitant respiratory viruses were most frequently identified in specimens collected from children with Delta infections (14 of 35 [40.0%]). Most participants (1224 of 1440 [85.0%]) completed a 14-day follow-up. Among 998 participants (69.3%) asked about their child’s SARS-CoV-2 vaccination status, 80 (8.0%) had received at least 1 dose, 816 (81.8%) had not received a dose, and 102 (10.2%) were unsure.

### Primary Outcome

**Figure 2.**
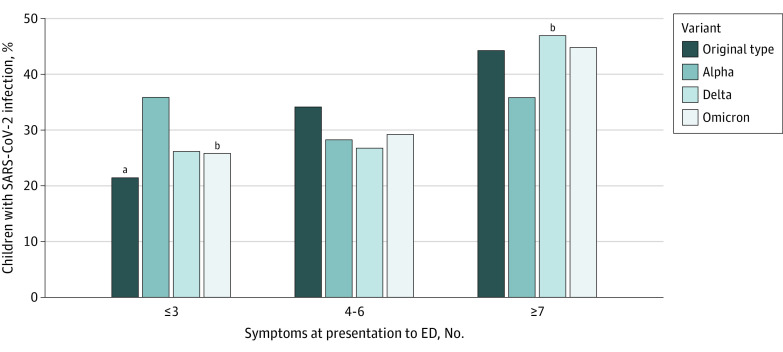
Number of Presenting Symptoms Reported by SARS-CoV-2 Variant Identified *P* values were adjusted for multiplicity using the Benjamini-Hochberg method. ED indicates emergency department. ^a^*P* = .001 compared with Alpha variant. ^b^*P* = .05 compared with Alpha variant.

Fever (1077 [75.1%]), cough (811 [56.6%]), and rhinorrhea (770 [53.8%]) were the most common individual symptoms (Table 1). Those infected with the original-type virus most often presented with abdominal pain (109 [26.7%]), ageusia (30 [7.3%]), anosmia (21 [5.1%]), and myalgias (80 [19.6%]). Participants with Alpha infection were least likely to be drowsy (77 [32.5%]) or to have conjunctivitis (7 [3.0%]), oral changes (12 [5.1%]), rhinorrhea (101 [42.6%]), and sore throat (38 [16.0%]). Those with Delta infection most often had conjunctivitis (32 [10.0%]), cough (197 [61.4%]), and upper respiratory tract symptoms (228 [71.0%]); those infected by Omicron were most often drowsy (212 [45.5%]), febrile (379 [81.0%]), and short of breath (154 [33.0%]). Patients with the Alpha variant had the fewest total number of symptoms (median, 5 [IQR, 3.0-8.0]). Among Alpha-infected participants, only 85 of 237 (35.9%) reported at least 7 symptoms (difference Delta vs Alpha, 11.2% [95% CI, 3.0%-19.4%]) (Table 1 and Figure 2).

**Figure 3.**
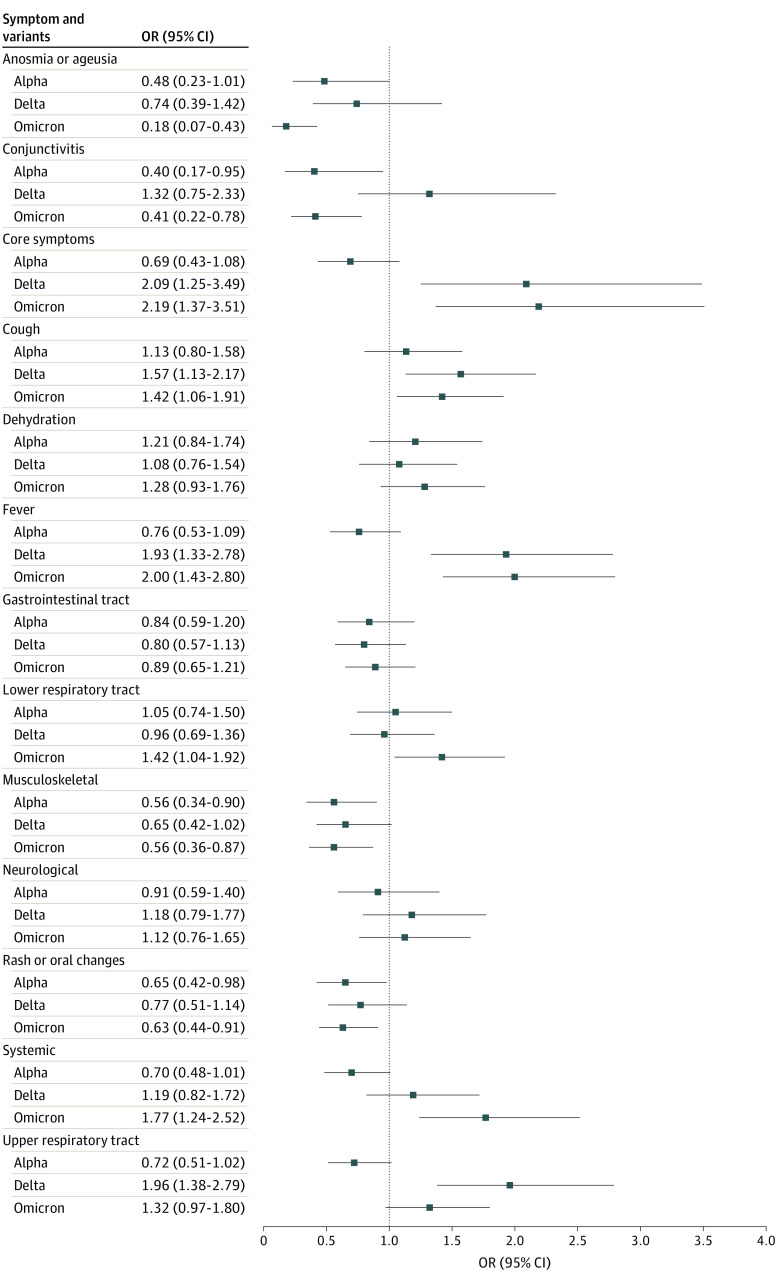
Mixed-Effect Binary Logistic Regression Model Examining the Odds of Experiencing a Given Symptom or Symptom Group by Variant of Concern Original-type SARS-CoV-2 serves as the reference group. OR indicates odds ratio.

In the multivariable model, Omicron and Delta infections were associated with fever (ORs, 2.00 [95% CI, 1.43-2.80] and 1.93 [95% CI, 1.33-2.78], respectively) and cough (ORs, 1.42 [95% CI, 1.06-1.91] and 1.57 [95% CI, 1.13-2.17], respectively) (Figure 3 and eTable 1 in Supplement 1). Delta infection was associated with the reporting of upper respiratory tract symptoms (OR, 1.96 [95% CI, 1.38-2.79]), while Omicron infection was associated with lower respiratory tract symptoms (OR, 1.42 [95% CI, 1.04-1.92]) and systemic symptoms (OR, 1.77 [95% CI, 1.24-2.52]). Musculoskeletal symptoms were more strongly associated with the original-type virus than any VoC (OR for Alpha, 0.56 [95% CI, 0.34-0.90]; OR for Delta, 0.65 [95% CI, 0.42-1.02]; and OR for Omicron, 0.56 [95% CI, 0.36-0.87]). Anosmia or ageusia and rash or oral changes were less strongly associated with the Alpha (ORs, 0.48 [95% CI, 0.23-1.01] and 0.65 [95% CI, 0.42-0.98], respectively) and Omicron (ORs, 0.18 [95% CI, 0.07-0.43] and 0.63 [95% CI, 0.44-0.91], respectively) strains relative to the original type.

### Secondary Outcomes

Most study participants (1277 of 1435 [89.0%]) experienced a core COVID-19 symptom. These symptoms were most common among participants with Omicron infection (434 of 468 [92.7%]) and lowest among those with Alpha infection (195 of 237 [82.3%]; difference, 10.5% [95% CI, 5.1%-15.9%]) (Table 1).

Patients infected with Omicron, compared with Alpha and Delta, were more likely to have chest radiography performed (Omicron vs Alpha difference, 9.6% [95% CI, 4.2%-15.0%]; Omicron vs Delta difference, 9.7% [95% CI, 4.7%-14.8%]) and more likely to receive intravenous fluids (Omicron vs Alpha difference, 9.5% [95% CI, 5.1%-14.0%]; Omicron vs Delta difference, 5.6% [95% CI, 1.0%-10.2%]). Compared with children with other variant infections, those with Omicron infection were more likely to receive corticosteroids (Omicron vs original-type difference, 8.3% [95% CI, 3.8%-12.8%]; Omicron vs Alpha difference, 5.7% [95% CI, 0.3%-11.1%]; and Omicron vs Delta difference, 7.9% [95% CI, 3.2%-12.7%]). In addition, participants with Omicron infection were more likely than those with Delta infection to have additional ED visits (difference, 8.8% [95% CI, 3.5%-14.1%]). Overall, 164 children (11.4%) were admitted to the hospital and 9 (0.6%) were admitted to the ICU, with no differences between variants (Table 2).

**Table 2.** Clinical Course of SARS-CoV-2–Infected Children by Variant of Concern at Index ED Visit or Within 14-Day Follow-up Period

Course	Variant, No./total No. (%)					*P* value
	All patients (N = 1440)	Original type (n = 409)	Alpha (n = 242)	Delta (n = 321)	Omicron (n = 468)	
Outcomes						
Admitted to hospital	164/1440 (11.4)	52/409 (12.7)	18/242 (7.4)	33/321 (10.3)	61/468 (13.0)	.23
Admitted to ICU	9/1440 (0.6)	3/409 (0.7)	3/242 (1.2)	2/321 (0.6)	1/468 (0.2)	.50
Any revisit	376/1250 (30.1)	120/372 (32.3)	68/214 (31.8)	72/267 (27.0)	116/397 (29.2)	.53
ED revisit	192/1250 (15.4)	53/372 (14.2)	37/214 (17.3)	27/267 (10.1)	75/397 (18.9)	.04
Hospitalized at revisit	29/1440 (2.0)	12/409 (2.9)	3/242 (1.2)	5/321 (1.6)	9/468 (1.9)	.53
Died	0/407	0/242	0/317	0/465	0/407	NA
Chest radiography and treatments						
Chest radiography	228/1433 (15.9)	69/407 (17.0)	27/242 (11.2)	35/317 (11.0)	97/467 (20.8)	.004
Intravenous fluids	166/1430 (11.6)	50/406 (12.3)	14/242 (5.8)	31/319 (9.7)	71/463 (15.3)	.01
Oxygen	22/1428 (1.5)	5/406 (1.2)	1/242 (0.4)	7/318 (2.2)	9/462 (1.9)	.45
Antibiotics	213/1433 (14.9)	62/406 (15.3)	27/242 (11.2)	54/319 (16.9)	70/466 (15.0)	.45
Corticosteroids	180/1429 (12.6)	38/405 (9.4)	29/242 (12.0)	31/318 (9.7)	82/464 (17.7)	<.001
Mechanical ventilation^a^	6/1427 (0.4)	1/406 (0.2)	1/242 (0.4)	2/317 (0.6)	2/462 (0.4)	.94

Abbreviations: ED, emergency department; ICU, intensive care unit; NA, not applicable.

^a^ Includes invasive or noninvasive.

### Sensitivity Analysis

Children with VoC testing performed did not differ in their symptom profile or outcomes compared with those who had their VoC inferred. The exceptions included days of illness among those with Delta infection (median, 3.0 [IQR, 1.5-5.0] days among those tested vs 4.0 [IQR, 2.0-6.0] days among those inferred; *P* = .03) and the presence of irritability among children with Alpha infection (76 of 154 [49.4%] among those tested vs 21 of 83 [25.3%] among those inferred; *P* = .02) (eTables 2 and 3 in Supplement 1).

## Discussion

Among children positive for SARS-CoV-2 who presented for care in pediatric EDs across Canada, symptom profiles differed based on etiologic variants. Children with the Alpha variant had the fewest number of presenting symptoms, while those with the Delta variant had the greatest number of symptoms. While the latter group of children were most likely to report conjunctivitis and upper respiratory tract symptoms, they were also most likely to have codetection of an additional virus. Although the characteristics of presenting symptoms changed as the SARS-CoV-2 virus evolved, unlike in adults where mortality declined in subsequent waves,^25^ the proportions of infected children experiencing undesirable outcomes in our study remained stable.

Children with Omicron infection are nearly twice as likely to experience fever as those with original-type SARS-CoV-2 infection and 1.5 times more likely to report a cough. Although Omicron has been associated with croup,^13^ we did not find that upper respiratory tract symptoms were more common in children with Omicron infection when compared with other variants. However, croup and stridor were not specific data fields in our study, which may have led to underreporting of upper respiratory tract symptoms. Our results show that children with Omicron infection reported lower respiratory tract symptoms (eg, shortness of breath, chest pain, wheezing, or sputum) more frequently than was reported with any of the other variants. Additionally, systemic manifestations such as apnea, irritability, lethargy, and drowsiness were more commonly reported by children with Omicron infection than among those with earlier variants. Although anosmia and ageusia were rare complaints in participants with Omicron infection,^26^ participants infected by Omicron most commonly had core COVID-19 symptoms.^19^ While several reports described Omicron as being responsible for less severe disease than prior variants,^27^ particularly among adults,^28,29^ we found that children with Omicron infection received more interventions and were more likely to experience ED revisits. Our findings are not unique, as they align with other pediatric studies that report higher pediatric hospitalization rates^30,31,32^ during the Omicron period.

Similar to earlier studies,^33,34^ fever and cough were the most common presenting symptoms in our cohort irrespective of infecting variant, being reported by over half of the children in our cohort. This is confounded, however, by the fact that fever and/or cough were commonly used indications for SARS-CoV-2 testing in study institutions. Our results align with previous reports of how symptom patterns evolved with the Delta and Omicron variants,^35^ both of which led to fever, congestion, and cough becoming more common presenting symptoms, while the prevalence of myalgias, anosmia, and ageusia declined. This may reflect changes in testing patterns over time, with hospitals moving from a “test all” approach to a more selective strategy testing only those who are more unwell or with core COVID-19 symptoms. Further, the higher prevalence of respiratory symptoms in the Omicron and Delta groups may reflect coinfections that, as we report, became more common later in the pandemic as seasonal respiratory viruses resurfaced.^36^

Children infected with the Omicron strain consumed significant resources, being most likely to have chest radiography performed, to have intravenous fluids and corticosteroids administered, and to revisit the ED within 14 days. This may reflect several factors, including the selective testing of more unwell children, changes in parental thresholds for seeking ED care, higher rates of coinfection, more lower respiratory tract and systemic symptoms, and the younger age of children with Omicron infection.

Although the overall proportions of participants experiencing severe disease was lower than has been previously reported,^37^ they are consistent with previous reports from our hospital network.^38^ This may reflect the fact that our study did not capture children transported from referral hospitals directly to our ICU and that in Canada, the EDs often served as a SARS-CoV-2 testing location due to testing capacity limitations elsewhere. Nonetheless, the rates of hospitalization,^39^ ICU admission, and total health care provider revisits did not differ between variants. Thus, unlike in adults, it does not appear that children are being less severely affected by emerging variants, and understanding the clinical presentation of COVID-19 in children is needed to design therapeutic trials on this population. Moreover, as the pediatric COVID-19 clinical phenotype shift occurs over time, clinicians should remain alert to its possible presence, test when clinically indicated, and treat when appropriate (eg, corticosteroids in children hospitalized with COVID-19–associated pneumonia).^40^

### Limitations

This study has several limitations. First, only one-third of our cohort had VoC testing performed, and the remaining two-thirds had the most likely variant inferred based on the dominant strain in Canada at the time of testing; therefore, our variant classification is uncertain. Additionally, the proportion imputed differed between variants, with most of the original-type virus and Omicron VoC being imputed, while most of the Alpha and Delta VoC were confirmed. Although there is a higher chance of misclassification error among the original-type and Omicron variants and thus a higher chance of incorrectly attributing symptoms or outcomes to those variants, there were minimal VoC cases during the original-type period, and once the Omicron VoC became predominant, the presence of non-Omicron cases declined sharply. While we compared tested vs inferred groups and found few statistically significant differences, in several instances, the difference in proportions of participants with a particular symptom may be clinically meaningful despite lacking sufficient power to achieve statistical significance. Additionally, we were unable to separate subvariants of Omicron for analysis despite there being recognized differences in clinical profiles.^41^

Selection bias may have affected which children were included in our cohort, as children with symptoms associated with COVID-19 infection were more likely to be tested. Symptoms were self-reported by the children and/or caregivers; therefore, the presence of subjective symptoms such as anosmia or ageusia, myalgias, and headache may have been difficult to ascertain, particularly in preverbal children. Additionally, we cannot exclude the possibility that viral coinfection influenced the symptoms reported; although this occurred in 15% of participants in our study who were tested, we cannot extrapolate this to our broader cohort, as only 16% of participants had respiratory virus testing performed in addition to SARS-CoV-2 testing.

As these variants were not circulating simultaneously, the variation we report in symptoms occurred in the context of varying levels of natural and vaccine-induced immunity and thus cannot be attributed solely to the variant. Furthermore, given the low overall vaccination rate in our cohort at the time of enrollment, we could not assess the impact of vaccination on presenting symptoms. Finally, our cohort represents only children with symptoms severe enough to warrant presentation to the ED; therefore, the profiles we describe reflect those of our study population and cannot be extrapolated to all pediatric patients. Regardless, our results provide a unique perspective on the changing symptoms and disease severity among children with COVID-19 over time and characterize the clinical presentation of the current strain, Omicron, which has led to significant use of ED resources.

## Conclusions

The findings of this cohort study suggest that children with Omicron infection were more likely to present with fever, lower respiratory tract symptoms, and systemic manifestations than those with earlier variants. These findings highlight the importance of remaining vigilant to evolving clinical presentations and testing patients when clinically indicated. Importantly, although the characteristics of presenting symptoms have changed as the SARS-CoV-2 virus has evolved, unlike in adults, the proportions of children with COVID-19 experiencing undesirable outcomes have remained stable.

## Notes

Supplement 1. **eTable 1.** Results of Mixed-Effect Binary Logistic Regression Model, Examining the Odds of Experiencing a Given Symptom or Symptom Group by Variant of Concern **eTable 2.** Comparison of Symptom Profiles by Method of Variant of Concern Assignment (Reported vs Imputed) **eTable 3.** Comparison of Clinical Course and Outcomes at Index ED Visit or Within 14-Day Follow-up Period, by Method of Variant of Concern Assignment (Reported vs Imputed) **eFigure.** Chronological Depiction of Timing of Identification of Variants and Proportional Breakdown of Identification Through Testing or Imputation Based on Known Dominant Strains

Supplement 2. **Nonauthor Collaborators. **Pediatric Emergency Research Canada Collaborators

Supplement 3.
Data Sharing Statement
