# Supplementary material for: Comparison of Symptoms Associated With SARS-CoV-2 Variants Among Children in Canada
Source: JAMA Netw Open. 2023 Mar 9;6(3):e232328. doi: 10.1001/jamanetworkopen.2023.2328 (PMC9999248; doi:10.1001/jamanetworkopen.2023.2328)
Supplement: Supplement 1. — eTable 1. Results of Mixed-Effect Binary Logistic Regression Model, Examining the Odds of Experiencing a Given Symptom or Symptom Group by Variant of Concern eTable 2. Comparison of Symptom Profiles by Method of Variant of Concern Assignment (Reported vs Imputed) eTable 3. Comparison of Clinical Course and Outcomes at Index ED Visit or Within 14-Day Follow-up Period, by Method of Variant of Concern Assignment (Reported vs Imputed) eFigure. Chronological Depiction of Timing of Identification of Variants and Proportional Breakdown of Identification Through Testing or Imputation Based on Known Dominant Strains [file jamanetwopen-e232328-s001.pdf]

Supplementary Online Content

Sumner MW, Xie J, Zemek R, et al; Pediatric Emergency Research Canada (PERC) COVID Study Group. Comparison of symptoms associated with SARS-CoV-2 variants among children in Canada. *JAMA Netw Open*. 2023;6(3):e232328. doi:10.1001/jamanetworkopen.2023.2328

eTable 1. Results of Mixed-Effect Binary Logistic Regression Model, Examining the Odds of Experiencing a Given Symptom or Symptom Group by Variant of Concern
eTable 2. Comparison of Symptom Profiles by Method of Variant of Concern Assignment (Reported vs Imputed)
eTable 3. Comparison of Clinical Course and Outcomes at Index ED Visit or Within 14-Day Follow-up Period, by Method of Variant of Concern Assignment (Reported vs Imputed)
eFigure. Chronological Depiction of Timing of Identification of Variants and Proportional Breakdown of Identification Through Testing or Imputation Based on Known Dominant Strains

This supplementary material has been provided by the authors to give readers additional information about their work.

© 2023 Sumner MW et al. *JAMA Network Open*.

eTable 1. Results of Mixed-Effect Binary Logistic Regression Model, Examining the Odds of Experiencing a Given Symptom or Symptom Group by Variant of Concern

	Variant of Concern (VoC				Sex		Age			Illness Duration			Hospitalized	
	**Omicron**	**Delta**	**Alpha**	**Original type**	**Female**	**Male**	**≥ 4 yrs**	**1-3 yrs**	**< 1 yr**	**≥ 4 days**	**2-3 days**	**≤1 day**	**Yes**	**No**
**Core Symptom**	2.19 (1.37, 3.51)<br>P=0.001	2.09 (1.25, 3.49)<br>P=0.005	0.69 (0.43,1.08)<br>P=0.11	ref.	1.18 (0.83, 1.67)<br>P=0.35	ref.	0.45 (0.30, 0.68)<br>P<0.001	1.03 (0.63, 1.69)<br>P=0.89	ref.	1.24 (0.81, 1.88)<br>P = 0.32	1.34 (0.87, 2.07)<br>P = 0.18	ref.	0.57 (0.36, 0.93)<br>P = 0.08	ref.
**Fever**	2.00 (1.43, 2.80)<br>P<0.001	1.93 (1.33, 2.78)<br>P<0.001	0.76 (0.53, 1.09)<br>P=0.14	ref.	1.22 (0.95,1.57)<br>P=0.12	ref.	0.59 (0.44, 0.79)<br>P<0.001	1.23 (0.88, 1.73)<br>P=0.22	ref.	0.85 (0.63,1.16)<br>P=0.31	1.19 (0.86, 1.63)<br>P=0.29	ref.	0.74 (0.51, 1.08)<br>P=0.12	ref.
**Cough**	1.42 (1.06, 1.91)<br>P=0.02	1.57 (1.13, 2.17)<br>P=0.007	1.13 (0.80,1.58)<br>P=0.48	ref.	0.88 (0.71, 1.10)<br>P=0.26	ref.	0.57 (0.44, 0.74)<br>P<0.001	0.88 (0.66, 1.16)<br>P=0.36	ref.	2.56 (1.93, 3.38)<br>P<0.001	1.54 (1.17, 2.02)<br>P=0.002	ref.	0.67 (0.47, 0.94)<br>P=0.02	ref.
**Conjuncti-vitis**	0.41 (0.22, 0.78)<br>P=0.007	1.32 (0.75, 2.33)<br>P=0.33	0.40 (0.17, 0.95)<br>P=0.04	ref.	0.70 (0.44, 1.11)<br>P=0.13	ref.	0.70 (0.42, 1.16)<br>P=0.16	0.47 (0.25, 0.85)<br>P=0.01	ref.	1.43 (0.80, 2.58)<br>P=0.23	2.07 (1.18, 3.65)<br>P=0.01	ref.	1.19 (0.60, 2.38)<br>P=0.61	ref.
**Upper Respiratory Symptoms**	1.32 (0.97, 1.80)<br>P=0.07	1.96 (1.38, 2.79)<br>P<0.001	0.72 (0.51, 1.02)<br>P=0.07	ref.	1.06 (0.84, 1.33)<br>P=0.62	ref.	0.65 (049, 0.85)<br>P=0.002	0.84 (0.62, 1.12)<br>P=0.24	ref.	2.50 (1.88, 3.34)<br>P<0.001	2.04 (1.53, 2.70)<br>P<0.001	ref.	0.62 (0.43, 0.88)<br>P=0.008	ref.
**Lower Respiratory Symptoms**	1.42 (1.04, 1.92)<br>P=0.03	0.96 (0.69, 1.36)<br>P=0.83	1.05 (0.74, 1.50)<br>P=0.77	ref.	0.89 (0.71, 1.12)<br>P=0.31	ref.	0.94 (0.72, 1.24)<br>P=0.68	0.98 (0.73, 1.31)<br>P=0.88	ref.	1.83 (1.38, 2.42)<br>P<0.001	1.48 (1.12, 1.97)<br>P=0.007	ref.	0.96 (0.66, 1.40)<br>P=0.85	ref.
**GI**	0.89 (0.65, 1.21)<br>P=0.45	0.80 (0.57, 1.13)<br>P=0.21	0.84 (0.59, 1.20)<br>P=0.34	ref.	1.37 (1.09, 1.72)<br>P=0.007	ref.	1.54 (1.18, 2.02)<br>P=0.002	1.57 (1.18, 2.09)<br>P=0.002	ref.	1.79 (1.35, 2.37)<br>P<0.001	1.77 (1.34, 2.35)<br>P<0.001	ref.	1.54 (1.07, 2.23)<br>P=0.008	ref.
**Neurologic**	1.12 (0.76, 1.65)<br>P=0.57	1.18 (0.79, 1.77)<br>P=0.41	0.91 (0.59, 1.40)<br>P=0.66	ref.	1.39 (1.05, 1.85)<br>P=0.02	ref.	33.65 (19.69, 57.49)<br>P<0.001	6.09 (3.46, 10.73)<br>P<0.001	ref.	1.50 (1.06, 2.14)<br>P=0.02	1.26 (0.88, 1.82)<br>P=0.21	ref.	1.09 (0.68, 1.73)<br>P=0.91	ref.
**MSK**	0.56 (0.36, 0.87)<br>P=0.009	0.65 (0.42, 1.02)<br>P=0.06	0.56 (0.34, 0.90)<br>P=0.02	ref.	1.59 (1.16, 2.18)<br>P=0.004	ref.	12.87 (7.55, 21.94)<br>P<0.0001	3.77 (2.12, 6.72)<br>P<0.001	ref.	1.69 (1.14, 2.51)<br>P=0.01	1.59 (1.06, 2.39)<br>P=0.02	ref.	0.68 (0.38, 1.21)<br>P=0.19	ref.
**Rash, oral**	0.63 (0.44, 0.91)	0.77 (0.51, 1.14)	0.65 (0.42, 0.98)	ref.	1.09 (0.83, 1.43)	ref.	0.84 (0.61, 1.16)	0.65 (0.46, 0.92)	ref.	1.69 (1.21, 2.35)	1.18 (0.84, 1.67)	ref.	0.93 (0.59, 1.46)	ref.

© 2023 Sumner MW et al. *JAMA Network Open*.

**changes,**	P=0.01	P=0.19	P=0.04		P=0.54		P=0.30	P=0.01		P=0.002	P=0.34		P=0.75	
GU/dehydration	1.28 (0.93, 1.76)<br><br>P=0.14	1.08 (0.76, 1.54)<br><br>P=0.67	1.21 (0.84, 1.74)<br><br>P=0.31		1.34 (1.06, 1.70)<br><br>P=0.01		0.72 (0.54, 0.95)<br><br>P=0.02	1.03 (0.76, 1.38)<br><br>P=0.86		1.42 (1.06, 1.91)<br><br>P=0.02	1.50 (1.12, 2.01)<br><br>P=0.007		1.76 (1.20, 2.57)<br><br>P=0.004	
Systemic	1.77 (1.24, 2.52)<br><br>P=0.002	1.19 (0.82, 1.72)<br><br>P=0.37	0.70 (0.48, 1.01)<br><br>P=0.06		1.05 (0.81, 1.35)<br><br>P=0.71		0.60 (0.44, 0.81)<br><br>P=0.001	0.86 (0.62, 1.2)<br><br>P=0.38		1.37 (1.00, 1.87)<br><br>P=0.05	1.36 (0.99, 1.87)<br><br>P=0.06		0.91 (0.61, 1.36)<br><br>P=0.65	
Anosmia/ageusia	0.18 (0.07, 0.43)<br><br>P<0.001	0.74 (0.39, 1.42)<br><br>P=0.37	0.48 (0.23, 1.005)<br><br>P=0.05		1.5 (0.91, 2.46)<br><br>P=0.11		69.63 (9.5, 510.47)<br><br>P<0.001	12.89 (1.66, 100.0)<br><br>P=0.02		1.98 (1.04, 3.77)<br><br>P=0.04	2.09 (1.08, 4.04)<br><br>P=0.03		0.76 (0.31, 1.85)<br><br>P=0.54	

Data are adjusted for age, sex, number of days of illness at ED presentation, and hospitalization status. Data are reported as odds ratios (95% CI).
a - Symptoms were grouped as follows: upper respiratory = rhinorrhea and/or sore throat, lower respiratory = shortness of breath, pleuritic or non-pleuritic chest pain, wheezing and/or sputum production, GI = abdominal pain, vomiting, diarrhea and/or anorexia, neurologic = headache and/or seizures, MSK= joint and/or muscle pain, dehydration = decreased urine output and/or refusal to drink, systemic = irritability, lethargy, apnea and/or drowsiness, rash/oral changes = rash to skin, hands, feet and/or oral or extremity changes.

© 2023 Sumner MW et al. *JAMA Network Open*.

eTable 2. Comparison of Symptom Profiles by Method of Variant of Concern Assignment (Reported vs Imputed)

	Original-type Imputed (n=409)	Original-type Reported (n=0)	P-value[a]	Alpha Imputed (n=84[c])	Alpha Reported (n=158[d])	P-value[a]	Delta Imputed (n=144)	Delta Reported (n=177)	P-value[a]	Omicron Imputed (n=422[e])	Omicron Reported (n=46[e])	P-value[a]
**Age, years, median (IQR)**	1.3 (0.6, 6.0)	N/A	N/A	2.0 (1.0, 7.0)	1.7 (0.8, 7.0)	0.95	2.0 (0.9, 7.0)	3.0 (0.8, 9.0)	0.67	1.0 (0.3, 6.0)	3.0 (1.0, 7.0)	0.13
**Age groups**		N/A	N/A			0.93			0.28			0.13
< 1 year	135 (33.0)			18 (21.4)	47 (29.7)		38 (26.4)	49 (27.7)		190 (45.0)	10 (21.7)	
1 to 3 years	133 (32.5)			33 (39.3)	55 (34.8)		50 (34.7)	43 (24.3)		97 (23.0)	15 (32.6)	
≥ 4 years	141 (34.5)			33 (39.3)	56 (35.4)		56 (38.9)	85 (48.0)		135 (32.0)	21 (45.7)	
**Sex, male, n (%)**	227 (55.5)	N/A	N/A	51 (60.7)	97 (61.4)	0.99	80 (55.6)	96 (54.2)	0.94	221 (52.4)	29 (63.0)	0.47
**Days of illness, median (IQR)**	2.0 (1.0, 4.0)	N/A	N/A	2.0 (1.0, 5.0)	2.0 (0.5, 4.0)	0.55	4.0 (2.0, 6.0)	3.0 (1.5, 5.0)	0.03	3.0 (1.0, 4.0)	2.0 (0.5, 3.0)	0.27
**Days of illness**		N/A	N/A			0.93			0.12			0.37
≤1 day	158 (38.6)			31 (37.3)	69 (44.8)		24 (16.7)	44 (24.9)		126 (29.9)	21 (45.7)	
2 to 3 days	130 (31.8)			23 (27.7)	44 (28.6)		39 (27.1)	62 (35.0)		158 (37.4)	15 (32.6)	
≥ 4 days	121 (29.6)			29 (34.9)	41 (26.6)		81 (56.3)	71 (40.1)		138 (32.7)	10 (21.7)	
**Core COVID Symptoms[b]**	55 (13.4)	N/A	N/A	68 (81.9)	127 (82.5)	0.99	130 (90.3)	164 (92.7)	0.71	390 (92.4)	44 (95.7)	0.77
Fever	290 (70.9)	N/A	N/A	50 (60.2)	106 (68.8)	0.93	106 (73.6)	146 (82.5)	0.20	341 (80.8)	38 (82.6)	0.94

© 2023 Sumner MW et al. *JAMA Network Open*.

Cough	211 (51.6)	N/A	N/A	43 (51.8)	86 (55.8)	0.98	90 (62.5)	107 (60.5)	0.92	245 (58.2)	29 (64.4)	0.77
Conjunctivitis	31 (7.6)	N/A	N/A	2 (2.4)	5 (3.2)	0.99	9 (6.3)	23 (13)	0.21	17 (4.0)	1 (2.2)	0.99
**Upper Respiratory Symptoms**	241 (58.9)	N/A	N/A	42 (50.6)	77 (50)	0.99	101 (70.1)	127 (71.8)	0.94	259 (61.5)	29 (64.4)	0.89
Rhinorrhea	215/408 (52.7)	N/A	N/A	36 (43.4)	65 (42.2)	0.99	89 (61.8)	109 (61.6)	0.99	234 (55.6)	22 (48.9)	0.77
Sore throat	76 (18.6)	N/A	N/A	14 (16.9)	24 (15.6)	0.99	33 (22.9)	58 (32.8)	0.20	92 (21.9)	18 (40.0)	0.13
**Lower Respiratory Symptoms**	145 (35.5)	N/A	N/A	33 (39.8)	52 (33.8)	0.93	45 (31.3)	67 (37.9)	0.45	177 (42)	26 (57.8)	0.30
Shortness of breath	88 (21.5)	N/A	N/A	23 (27.7)	30 (19.5)	0.93	24 (16.7)	44 (24.9)	0.24	131 (31.1)	23 (51.1)	0.13
Chest pain	38 (9.3)	N/A	N/A	8 (9.6)	11 (7.1)	0.96	10 (6.9)	13 (7.3)	0.99	22 (5.2)	2 (4.4)	0.99
Chest pain (pleuritic)	14 (3.4)	N/A	N/A	3 (3.6)	3 (1.9)	0.93	2 (1.4)	4 (2.3)	0.92	12 (2.9)	2 (4.4)	0.83
Wheezing	55 (13.4)	N/A	N/A	15 (18.1)	14 (9.1)	0.55	24 (16.7)	20 (11.3)	0.38	70 (16.6)	9 (20)	0.82
Sputum	38 (9.3)	N/A	N/A	7 (8.4)	15 (9.7)	0.99	20 (13.9)	20 (11.3)	0.75	57 (13.5)	4 (8.9)	0.77
**GI**	272 (66.5)	N/A	N/A	56 (67.5)	90 (58.4)	0.93	94 (65.3)	107 (60.5)	0.64	254 (60.3)	33 (73.3)	0.39
Abdominal pain	109 (26.7)	N/A	N/A	21 (25.3)	29 (18.8)	0.93	27 (18.8)	35 (19.8)	0.94	81 (19.2)	11 (24.4)	0.77
Vomiting	114 (27.9)	N/A	N/A	28 (33.7)	34 (22.1)	0.55	38 (26.4)	42 (23.7)	0.85	110 (26.1)	11 (24.4)	0.97
Diarrhea	107 (26.2)	N/A	N/A	21 (25.3)	27 (17.5)	0.93	30 (20.8)	33 (18.6)	0.87	86 (20.4)	6 (13.3)	0.72
Anorexia	155 (37.9)	N/A	N/A	37 (44.6)	58 (37.7)	0.93	59 (41)	71 (40.1)	0.98	148 (35.2)	22 (48.9)	0.37
**Neurologic**	100 (24.4)	N/A	N/A	19 (22.9)	39 (25.3)	0.99	34 (23.6)	65 (36.7)	0.11	91 (21.6)	15 (33.3)	0.37
Headache	93 (22.7)	N/A	N/A	17 (20.5)	36 (23.4)	0.99	31 (21.5)	58 (32.8)	0.17	86 (20.4)	12 (26.7)	0.72

© 2023 Sumner MW et al. *JAMA Network Open*.

Seizures	10 (2.4)	N/A	N/A	3 (3.6)	3 (1.9)	0.93	4 (2.8)	8 (4.5)	0.84	10 (2.4)	3 (6.7)	0.42
**MSK**	83 (20.3)	N/A	N/A	12 (14.5)	21 (13.6)	0.99	16 (11.1)	39 (22.0)	0.11	48 (11.4)	7 (15.6)	0.77
Muscle pain	80 (19.6)	N/A	N/A	12 (14.5)	21 (13.6)	0.99	15 (10.4)	34 (19.2)	0.18	43 (10.2)	6 (13.3)	0.77
Joint pain	50 (12.2)	N/A	N/A	6 (7.2)	16 (10.4)	0.93	13 (9.0)	29 (16.4)	0.20	29 (6.9)	6 (13.3)	0.42
**Rash or oral changes**	105 (25.7)	N/A	N/A	13 (15.7)	30 (19.5)	0.95	20 (13.9)	38 (21.5)	0.24	76 (18.1)	8 (17.8)	0.99
Skin rash	52 (12.7)	N/A	N/A	6 (7.2)	19 (12.3)	0.93	12 (8.3)	16 (9.0)	0.94	33 (7.8)	6 (13.3)	0.63
Rash to hands	10 (2.4)	N/A	N/A	2 (2.4)	2 (1.3)	0.99	2 (1.4)	2 (1.1)	0.99	8 (1.9)	1 (2.2)	0.99
Rash to feet	14 (3.4)	N/A	N/A	3 (3.6)	6 (3.9)	0.99	1/143 (0.7)	3 (1.7)	0.87	6 (1.4)	1 (2.2)	0.77
Extremity changes	8 (2.0)	N/A	N/A	1 (1.2)	3 (1.9)	0.99	2 (1.4)	3 (1.7)	0.99	9 (2.1)	0 (0)	0.99
Oral changes	56 (13.7)	N/A	N/A	5 (6.0)	7 (4.5)	0.99	8 (5.6)	21 (11.9)	0.20	43 (10.2)	4 (8.9)	0.99
**Dehydration**	126 (30.8)	N/A	N/A	28 (33.7)	48 (31.2)	0.99	47 (32.6)	48 (27.1)	0.54	142 (33.7)	13 (28.9)	0.77
Decreased urine output	69 (16.9)	N/A	N/A	15 (18.1)	23 (14.9)	0.98	32 (22.2)	29 (16.4)	0.40	60 (14.3)	9 (20.0)	0.67
Refusal to drink	102 (24.9)	N/A	N/A	25 (30.1)	39 (25.3)	0.93	37 (25.7)	37 (20.9)	0.55	127 (30.2)	12 (26.7)	0.83
**Systemic**	273 (66.7)	N/A	N/A	40 (48.2)	96 (62.3)	0.55	82 (56.9)	117 (66.1)	0.26	295 (70.1)	34 (75.6)	0.77
Apnea	4 (1.0)	N/A	N/A	1 (1.2)	3 (1.9)	0.99	0 (0)	4 (2.3)	0.33	4 (1.0)	0 (0)	0.99
Irritable	194 (47.4)	N/A	N/A	21 (25.3)	76 (49.4)	0.02	58 (40.3)	68/176 (38.6)	0.94	186 (44.2)	27 (60.0)	0.30
Lethargic	174 (42.5)	N/A	N/A	29 (34.9)	57 (37.0)	0.99	54 (37.5)	66 (37.3)	0.99	194/420 (46.2)	19 (42.2)	0.83
Drowsy	147 (35.9)	N/A	N/A	28 (33.7)	49 (31.8)	0.99	49 (34.0)	70/176 (39.8)	0.54	193 (45.8)	19 (42.2)	0.83
**Loss of**	35 (8.6)	N/A	N/A	6 (7.2)	6 (3.9)	0.93	5 (3.5)	20 (11.3)	0.11	5 (1.2)	2 (4.4)	0.42

© 2023 Sumner MW et al. *JAMA Network Open*.

**smell/taste**												
Loss of smell	21 (5.1)	N/A	N/A	4 (4.8)	3 (1.9)	0.93	1 (0.7)	13 (7.3)	0.10	3 (0.7)	0 (0)	0.99
Loss of taste	30 (7.3)	N/A	N/A	6 (7.2)	5 (3.2)	0.93	5 (3.5)	17 (9.6)	0.20	4 (1.0)	2 (4.4)	0.42
**Number of symptoms at index visit, median (IQR)**	6.0 (4.0, 9.0)	N/A	N/A	5.0 (3.0, 8.0)	5.0 (3.0, 8.3)	0.99	6.0 (3.0, 9.0)	7.0 (3.0, 9.0)	0.38	6.0 (3.0, 9.0)	7.0 (4.0, 9.3)	N/A
**Number of symptoms at index visit**			N/A			0.99			0.52			0.63
≤ 3	88 (21.5)	N/A		31 (37.3)	54 (35.1)		39 (27.1)	45 (25.4)		112 (26.5)	9 (19.6)	
4 to 6	140 (34.2)	N/A		24 (28.9)	43 (27.9)		44 (30.6)	42 (23.7)		126 (29.9)	11 (23.9)	
≥ 7	181 (44.3)	N/A		28 (33.7)	57 (37)		61 (42.4)	90 (50.8)		184 (43.6)	26 (56.5)	

All data are n(%) unless otherwise indicated.
a Adjusted P-value for multiple comparisons via Benjamini-Hochberg method, within the set of tests
b >=1 of fever, cough, and/or loss of smell or taste
c missing data in one patient
d missing data in four patients
e missing data in one patient

© 2023 Sumner MW et al. *JAMA Network Open*.

eTable 3. Comparison of Clinical Course and Outcomes at Index ED Visit or Within 14-Day Follow-up Period, by Method of Variant of Concern Assignment (Reported vs Imputed)

	Original-type Imputed (n=389)	Original-type Reported (n=31)	P-value[b]	Alpha Imputed (n=54)	Alpha Reported (n=170)	P-value[b]	Delta Imputed (n=128)	Delta Reported (n=205)	P-value[b]	Omicron Imputed (n=430)	Omicron Reported (n=62)	P-value[b]
**OUTCOMES**												
**Admitted**	N/A	N/A	10/84 (11.9)	8/158 (5.1)	0.18	18/144 (12.5)	15/177 (8.5)	0.59	51/422 (12.1)	10/46 (21.7)	0.36	N/A
**Admitted to ICU**	N/A	N/A	1/84 (1.2)	2/158 (1.3)	0.99	0/144 (0)	2/177 (1.1)	0.67	1/422 (0.2)	0/46 (0)	0.99	N/A
**Any Revisit**	N/A	N/A	18/76 (23.7)	50/138 (36.2)	0.18	36/118 (30.5)	36/149 (24.2)	0.59	107/364 (29.4)	9/33 (27.3)	0.99	N/A
**Revisit to ED**	N/A	N/A	10/76 (13.2)	27/138 (19.6)	0.47	12/118 (10.2)	15/149 (10.1)	0.98	68/364 (18.7)	7/33 (21.2)	0.99	N/A
**Hospitalized at revisit**	N/A	N/A	1/84 (1.2)	2/158 (1.3)	0.99	1/144 (0.7)	4/177 (2.3)	0.67	7/422 (1.7)	2/46 (4.3)	0.66	N/A
**Mechanical ventilation[a]**	N/A	N/A	0/84 (0)	1/158 (0.6)	0.99	0/142 (0)	2/175 (1.1)	0.67	2/416 (0.5)	0/46 (0)	0.99	N/A
**Died**	N/A	N/A	0/84 (0)	0/158 (0)	N/A	0/142 (0)	0/175 (0)	N/A	0/419 (0)	0/46 (0)	N/A	N/A
**Chest x-ray/MANAGEMENT**												
**Chest x-ray**	69/407 (17.0)	N/A	N/A	13/84 (15.5)	14/158 (8.9)	0.29	21/142 (14.8)	14/175 (8.0)	0.56	89/421 (21.1)	8/46 (17.4)	0.99
**IV fluid**	50/406 (12.3)	N/A	N/A	9/84 (10.7)	5/158 (3.2)	0.13	15/142 (10.6)	16/177 (9.0)	0.77	60/417 (14.4)	11/46 (23.9)	0.36
**Oxygen**	5/406 (1.2)	N/A	N/A	0/84 (0)	1/158 (0.6)	0.99	4/143 (2.8)	3/175 (1.7)	0.77	8/416 (1.9)	1/46 (2.2)	0.99
**Antibiotics**	62/406	N/A	N/A	16/84 (19)	11/158 (7.0)	0.05	27/143 (18.9)	27/176 (15.3)	0.67	64/420 (15.2)	6/46 (13.0)	0.99

© 2023 Sumner MW et al. *JAMA Network Open*.

	(15.3)											
**Corticosteroids**	38/405 (9.4)	N/A	N/A	10/84 (11.9)	19/158 (12)	0.99	17/143 (11.9)	14/175 (8.0)	0.59	69/418 (16.5)	13/46 (28.3)	0.36
Co-detection of a respiratory pathogen	2/63 (3.2)	N/A	N/A	1/14 (7.1)	2/16 (12.5)	0.99	9/16 (56.3)	5/19 (26.3)	0.56	16/94 (17)	0/10 (0)	0.85

All data are n (%).
ED, emergency department; ICU, intensive care unit; IV, intravenous
a Invasive or non-invasive
b Adjusted P-value for multiple comparisons via Benjamini-Hochberg method, within the set of tests

© 2023 Sumner MW et al. *JAMA Network Open*.

eFigure. Chronological Depiction of Timing of Identification of Variants and Proportional Breakdown of Identification Through Testing or Imputation Based on Known Dominant Strains

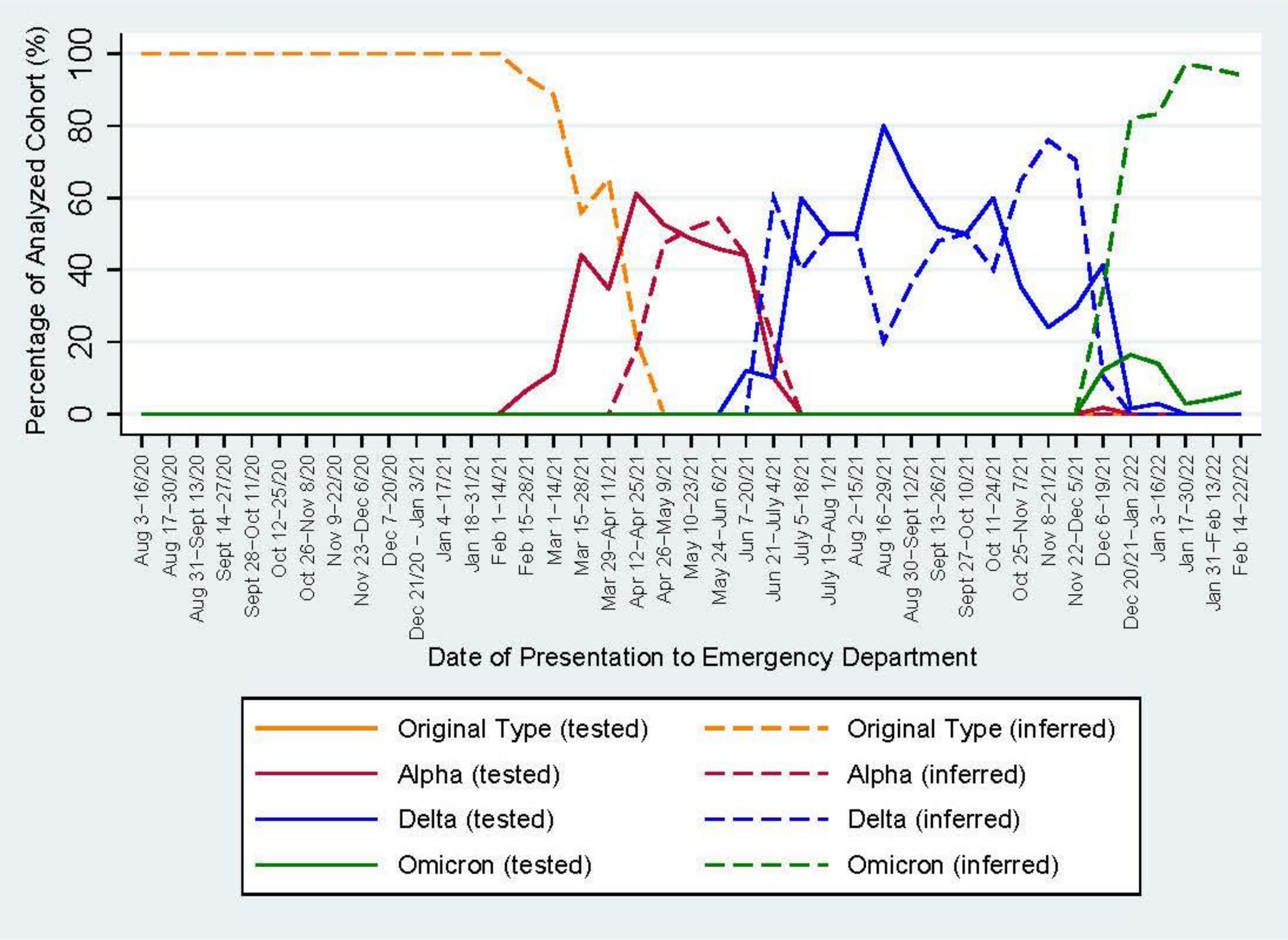

© 2023 Sumner MW et al. *JAMA Network Open*.
