# Supplementary material for: Comparison of Symptoms Associated With SARS-CoV-2 Variants Among Children in Canada
Source: JAMA Netw Open. 2023 Mar 9;6(3):e232328. doi: 10.1001/jamanetworkopen.2023.2328 (PMC9999248; doi:10.1001/jamanetworkopen.2023.2328)
Supplement: Supplement 2. — Nonauthor Collaborators. Pediatric Emergency Research Canada Collaborators [file jamanetwopen-e232328-s002.pdf]

Supplemental Online Content: Nonauthor Collaborators
*First name, last name, and suffix (if applicable) are required and will appear in PubMed.

*Group Name(s): Pediatric Emergency Research Canada (PERC) COVID Study Group							
***First Name and Middle Initial(s)**	***Last Name**	***Suffix (eg, Jr, III)**	Academic Degrees	Institution	Location (city, state/province, country)	Role or Contribution, eg, chair, principal investigator	Group (if more than 1 Group listed in the byline) and/or Subgroup (eg, Steering Committee)
Samina	Ali		MDCM	University of Alberta	Edmonton, AB, Canada	Study Oversight	
Mandi	Newton		PhD	University of Alberta	Edmonton, AB, Canada	Study Oversight	
Waleed	Alqurashi		MD	University of Ottawa	Ottawa, ON, Canada	Study Oversight	
Melissa	Lorenzo		MD	University of Toronto	Toronto, ON, Canada	Study Oversight	
Tyrus	Crawford		B.Soc.Sci	University of Ottawa	Ottawa, ON, Canada	Database Management	
Becky	Emerton		BSc	University of Calgary	Calgary, AB, Canada	Study Administration	
