# Supplementary material for: Comparison of Symptoms Associated With SARS-CoV-2 Variants Among Children in Canada
Source: JAMA Netw Open. 2023 Mar 9;6(3):e232328. doi: 10.1001/jamanetworkopen.2023.2328 (PMC9999248; doi:10.1001/jamanetworkopen.2023.2328)
Supplement: Supplement 3. — Data Sharing Statement [file jamanetwopen-e232328-s003.pdf]

# Data Sharing Statement

Sumner. Comparison of Symptoms Associated With SARS-CoV-2 Variants Among Children in Canada. *JAMA Netw Open*. Published March 09, 2023.
doi:10.1001/jamanetworkopen.2023.2328

**Data**
**Data available:** Yes
**Data types:** Deidentified participant data
**How to access data:** Data will be made available upon reasonable request and with appropriate ethics approvals and data sharing mechanisms in place.
**When available:** With publication

**Supporting Documents**
**Document types:** None

**Additional Information**
**Who can access the data:** Data will only be made available to researchers whose proposed use of the data has been approved.
**Types of analyses:** All data access requests will require specific research hypotheses or planned analyses.
**Mechanisms of data availability:** Data will be made available by the investigator after ethics approval has been obtained and signed data access agreements have been executed.
